# Medical Correspondence—Veratrum Viride

**Published:** 1854-07

**Authors:** 


### IOWA MEDICAL JOURNAL.

VOL. I. KEOKUK, IOWA, JULY, 1854. NO. 12.

### ORIGINAL COMMUNICATIONS.

### *Medical Correspondence—Veratrum Viride.*

May 16th, 1854.

Dr. Hard.—Dear Sir :

We received your very interesting article on Veratrum Viride, which will appear in the No. of our Journal soon to appear.

The American Helebore is now attracting much attention from the American Medical Profession, and if we are to take the declarations of the different writers who have used it, as an indication, it promises to rank prominently among our best therapeutic agents. Any thing therefore, which will confirm their experience and contribute to a further knowledge of the properties of this remedy, will be kindly received by the profession.

Previous to the receipt of your article, I had perused that of Dr. Norwood of S. C., which awakened in my mind the liveliest interest in, and an anxious desire to know more of its curative powers.—Among other diseases in which he found it highly useful, was one which the experience of every one has found to be highly formidable. I refer to *puerperal peritonitis.* The same experience teaches us that the time for successful action is early in the onset of the disease. And although there is much difference of opinion in the treatment of this grave affliction, yet an experience of many years has taught me to rely upon depletion as that upon which I could ground a hope of final success. As this agent (Helebore) possesses extraordinary sedative properties, it promises to become an important auxiliary to venesection, and possibly—nay, if its virtues have not been overstated, *probably* we may be able to rely in toto upon its sedative powers to control the pulse. This will be seen upon trial after its powers have been well tested in other inflammatory diseases in which there will be less hazard in delay, for when an inflammatory action takes place in the peritenæum, it sweeps onward like the fire in the prairies and equally destructive and affords no time for experimentation.

Although many years in practice, I have never used the *Veratrum Viride* in any form. Long since it sunk into the grave of obscurity, and neglect kept it entombed. It is true it was much vaunted by Hippocrates, who used it for every known disease and in every possible form. It was the remedy *lionized* in his day, the great panacea which had forever closed, and tightly sealed Pemdora's box. But *it* either became unfilial or *he* behaved toward it in a most unpaternal manner. His experience in its use convinced him that it possessed not only great, but dangerous powers, so much so that he became fearful of it, abandoned it and pronounced it deadly (*lethale*). Horace too lashed it with his keen satire and it gradually sunk into disuse, obscurity and neglect.

There is no one feature apparent in the habits of some medical men, which I regard as more reprehensible than that of mounting every *donkey* which comes ambling by them. Within a short time we have had a full *stud* of these "ponies." Some gratifying excursions are indulged in, the rider mounted on the speculum nitras argentum in hand to make an attack at the very gate way of the uterus and numerous have been the burnings and conflagrations with a *quantum sufficit* of unnecessary and uncalled for suffering. Then the probang proved another nag, jaded down in its frequent incursions into the laryngeal hall made more ample and spacious by frequent onslaughts upon its walls and other fixtures. Chloroform, the lightfooted and agile steed, after needlessly running many of its victims down, now and then plucking a green leaf by the way, is now jaded and fast approaching a slow and more consistent movement, proportioned to its ability and strength. Thus from one to another, the last always more highly vaunted than the one just discarded, and this too, in turn, to be kicked from their presence and anathematized as unsafe, unworthy of confidence, as useless and worthless, and thus dishonored, it sinks into obscurity.

Many new discoveries have been abandoned because used in milder cases when any thing would have succeeded or indeed when nothing was required, as for instance in the boasted cures of Homœopathy, but when they come to be tested in aggravated cases, would not meet their expectations. Although doubtless of value in some cases, they are nevertheless forever laid to one side, and not even used when there was an indication for their employment. I have a medical friend in my eye now, who, because *uva ursa* was extolled as superior to any other remedy in awakening uterine contraction, tried it, was disappointed in the result, as I have been, declared he would not use the article again in any case, as he believed it was of no more use than *dry straw.* This disgust arose from the bitter disappointment he was, under painful circumstances, compelled to endure, and yet he may find cases in which it would be eminently successful if resorted to. Others too have been laid aside because too highly vaunted and overdone, and because they did not fulfil *every* promise made for them, they were therefore fit for nothing. Care should be taken that too many properties may not be assigned to a simple article, for where little is expected, little will be required, and bitter disappointment is avoided. Others again have not been properly analysed and per consequence, improperly prepared, and therefore abandoned before experience has pointed out their *modus operandi* or their therapeutic powers. Suppose for instance, that chloroform had been wholly assigned the work of insensibility by inhalation, and from slovenly use, or criminal abuse, it falls into disrepute, the profession and humanity would, in that event, have been deprived of a valuable agent, because I regard its use by that mode as the least important of its therapeutic offices.

Any and every new remedy should be carefully analysed and its powers watched with the vigilance of an Argus' eye. It should be tried in a large number of cases of the character in which it first proved serviceable, before it is launched out into a wider sea in an experimental cruise against the whole fleet of distempers, in which event it is sure to wreck or founder. And yet had it not have been lost, it might have proved of infinite value, in its own legitimate way, to humanity in the relief of much human suffering.

Admit all this, and also that other fact, that the Helebore has been allowed to indulge in a long and undisturbed repose, is it any proof that it does not possess any claims to be ranked among other useful remedies known to science in their place in Materia Medica? The very fact of its antiquity gives it a claim to our consideration and investigation, and demands of us, in this day of progress, when science, from its greater perfectibility is able to give it a fairer and closer analysis, and when pathology is better understood,—a patient, impartial and thorough investigation of its powers. Doubtless Dr. Norwood errs in ascribing too many virtues to the article, but I would ask what remedy has not been overdone. One who stood prominent in the profession, once declared that he discovered one remedy, after a long experience, he could repose confidence in above all others, and this was mercury. All experience shows that this remedial agent, which has occupied so prominent a place in medical science, has been largely overdrawn and *overdone*, and has done more to weaken the confidence of the public in medical science, than all other circumstances combined. And who is there to deny that this agent possesses rare and undoubted remedial powers, although the abuse of it has given rise to a system whose basis was laid in the deep and immovable foundation of the prejudices of the populace. Others have been too much extolled also, for opium was regarded as almost a "divinity," and poetry made it the subject of its dulcet and enrapturing song.

For my part I deem the testimony in favor of Helebore sufficient to demand for it respectfull attention and a more extended trial of its capacities and powers, and because you have had much experience in its use, will you please communicate some additional facts. Will you inform me whether it grows in your vicinity, and if so could you furnish me with a specimen of the plant with root attached? Will you be kind enough to inform me also the *per cent* of the alcohol of which you make the saturated tincture and any thing else in relation to it which you might deem of interest to our readers and oblige,

Very respectfully, and truly, yours, &c.,

D. L. M'GUGIN.

---

AURORA, ILL., June 7, 1854.

Prof. M'GUGIN—

DEAR SIR: Your very valuable letter of the 16th May, was received after a long delay somewhere. And in reply I would say that not having the Veratrum Viride, I applied to G. L. Thompson, Druggist in Ottawa, who procured and prepared the tincture, which has been most successful in my hands. He informs me that it was made after the directions given in the U. S. Dispensatory. I have twice tried a tincture prepared by other druggists, which has failed to produce the desired effect, and I apprehend they made it from some other kinds of Veratrum. I am unable to say whether the plant grows in this vicinity or not, but I shall be on the "look out," and if I find it, you shall have a specimen. My partner, Dr. Young and myself are making a trial of its virtues in a number of chronic cases very different from any in which we have before used it, and the present indications are that it will prove highly useful.

One of the most formidable diseases with which we have to contend in the summer and autumn is dysentery. It frequently baffles the skill of our wisest heads, and reaps a harvest for death despite the efforts of the brightest ornaments of our profession. The following treatment I have lately tried, which proved so highly satisfactory that I think it worth a more extended trial.

℞ Oil Turpentine f ʒij,

Tinc. Opii. f ʒj,

Gum Acacia, White Sugar, } *a a* ʒiij,

Water f ʒij.

Rub thoroughly together to form an emulsion, to be given in teaspoonful doses every 3 or 4 hours, sufficiently often to control the discharges. If there is much tenesmus, increase the laudanum and use tincture of Veratrum Viride as in Pneumonia, to allay the inflammatory fever. We are well aware that the few cases in which we have used it, are insufficient to establish its reputation as a remedy in Dysentery, but we have strong hopes that it will prove a valuable auxiliary.

Cordially, your's,
A. HARD.

